# Probiotic DNA epigenetically modulates KDM4A degradation to mitigate airway allergy

**DOI:** 10.1016/j.isci.2025.114065

**Published:** 2025-11-19

**Authors:** Huanping Zhang, Xiaoyao Li, Mingxian Zhang, Xiaoxue Chen, Lihua Mo, Le Liu, Hanqing Zhang, Gaohui Wu, Qinmiao Huang, Yu Liu, Pingchang Yang

**Affiliations:** 1 Department of Allergy Medicine, Third Hospital of Shanxi Medical University, Shanxi Bethune Hospital, Shanxi Academy of Medical Sciences, Tongji Shanxi Hospital, Taiyuan 030032, China; 2 Department of Pulmonary and Critical Care Medicine, Third Hospital of Shanxi Medical University, Shanxi Bethune Hospital, Shanxi Academy of Medical Sciences, Tongji Shanxi Hospital, Taiyuan 030032, China; 3 State Key Laboratory of Respiratory Diseases Allergy Division at Shenzhen University and Institute of Allergy & Immunology, Shenzhen University School of Medicine, Shenzhen Key Laboratory of Allergy & Immunology, Shenzhen, China; 4 Department of Respirology and Allergy, Third Affiliated Hospital of Shenzhen University, Shenzhen 518055, China

**Keywords:** health sciences, molecular biology, immunology, immune response

## Abstract

Dysfunction of Type 1 regulatory T cells (Tr1 cells) is a key in airway allergy (AA), but mechanisms are unclear. This study explored if probiotic DNA restores Tr1 function in AA mice via USP14 and KDM4A. An AA mouse model (dust mite extracts) was used; techniques included flow cytometry (Tr1 function/Th2 polarization), ChIP, and ubiquitin immunoblotting (KDM4A, Il-10 promoter epigenetics). KDM4A-deficient CD4^+^ T cell mice showed spontaneous airway Th2 polarization (AA-like pathology). AA mice had impaired Tr1 immunosuppression, lower KDM4A at Il-10 promoter (correlating with reduced IL-10/Tr1 suppression), increased Il-10 promoter H3K9me3/hypermethylation, and reduced USP14. Probiotic DNA elevated USP14, restored KDM4A, enhanced Tr1 function, and reduced AA responses. In conclusion, reduced Il-10-promoter KDM4A drives Tr1 dysfunction in AA; probiotic DNA mitigates AA via USP14 upregulation (restoring KDM4A/Tr1 function), a potential therapeutic target.

## Highlights

• Probiotic DNA boosts USP14/KDM4A to restore Tr1 and alleviates airway allergy
• Dust-mite AA mice were analyzed by FACS, ChIP, and blot for KDM4A and Il-10 epigenetic marks
• KDM4A-null CD4^+^ T cells gained H3K9me3; AA Tr1 cells lost KDM4A, IL-10, and lost USP14
• Probiotic DNA raised USP14, reset KDM4A, revived Tr1, and eased AA

## Graphical abstract

**Figure:**
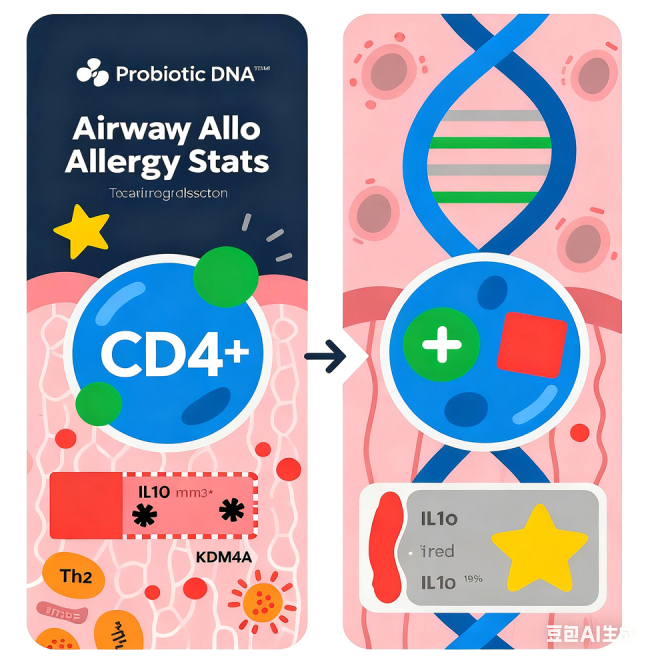

## Introduction

Dysfunctional immune regulation is increasingly recognized as a central feature in the pathogenesis of allergic diseases and other immune disorders. However, a comprehensive understanding of the precise underlying mechanisms remains elusive. The complex immune regulatory network within the body comprises various cell types, with regulatory B cells and regulatory T (Tregs) cells being particularly well-documented for their critical roles in maintaining immune homeostasis.^1^^,^^2^ Tregs encompass distinct subsets, including CD4^+^Foxp3^+^ Tregs and Type 1 regulatory T cells (Tr1 cells). A hallmark of Tr1 cells’ immune regulatory function is their primary reliance on interleukin-10 (IL-10), which acts to suppress the activities of other immune cells, thereby preventing excessive immune responses and self-injury. Notably, a consistent observation in individuals with airway allergy (AA) is a significant decrease in IL-10 production by Tr1 cells, which profoundly compromises their immune regulatory functions.^3^^,^^4^ Despite its known importance, the specific causes of this abnormal reduction in IL-10 expression in Tr1 cells within the context of AA are not fully understood.

Gene expression is intricately regulated by epigenetic mechanisms, primarily involving the methylation status of DNA.^5^ Specifically, hypomethylation generally promotes gene transcription, while hypermethylation typically suppresses it.^5^ Genes reside within chromosomes, where surrounding histones play a protective role, shielding the genetic material from unwanted disturbances.^6^ Demethylases are enzymes that facilitate histone demethylation, a process essential for allowing transcription factors to access and initiate gene transcription.^7^ Emerging evidence consistently links abnormal epigenetic conditions to the pathogenesis of various allergic diseases.^8^ The histone demethylase KDM4A, in particular, has been implicated in the pathogenesis of immune inflammation.^9^^,^^10^^,^^11^ Immune inflammation is frequently associated with the dysfunction of immune regulatory cells. However, whether actively modulating KDM4A activity can ameliorate dysfunctional immune regulation and its associated inflammatory conditions remains an area requiring further investigation.

There is a growing consensus regarding the immune regulatory functions of probiotics.^12^ Probiotics are live microorganisms, typically beneficial bacteria or yeasts, often administered to confer health benefits, particularly for gastrointestinal disorders. *Lactic-acid* bacteria, common components of the human microbiome and utilized for centuries in food production, are among the most frequently used probiotics. Other strains, such as *Bifidobacterium* and the yeast *Saccharomyces boulardii*, are also recognized for their probiotic effects.^13^ While probiotics are defined as live microorganisms that, when administered in adequate amounts, confer a health benefit on the host,^14^ the direct administration of live probiotics to the airways is often impractical or unsuitable. In this context, probiotic DNA, derived from beneficial bacteria, has emerged as a promising immunomodulatory agent. Probiotic DNA can interact with host cells, notably through Toll-like receptor 9 (TLR9) ligation, to influence gene expression and immune regulation.^15^ In the specific context of AAs, probiotic DNA has the potential to suppress the degradation of key epigenetic regulators like KDM4A,^16^ thereby contributing to the reduction of allergic inflammation. This unique characteristic suggests that probiotic DNA could serve as a novel therapeutic strategy with broad potential clinical applications.^17^^,^^18^^,^^19^ Therefore, we hypothesize that probiotic DNA may improve the function of Tr1 cells through modulating the activities of KDM4A. The present study aims to delineate the mechanism by which KDM4A deficiency leads to Tr1 cell dysfunction in AA and to investigate whether *Lactobacillus rhamnosus*-derived DNA (LR-DNA) can rescue Tr1 function. We reveal a decrease in KDM4A in the *Il-10* promoter of airway Tr1 cells in AA mice and demonstrate its correlation with the AA response.

## Results

### Ablation of the *Kdm4a* gene impairs the immunosuppressive function of Tr1 cells

**Figure 1:**
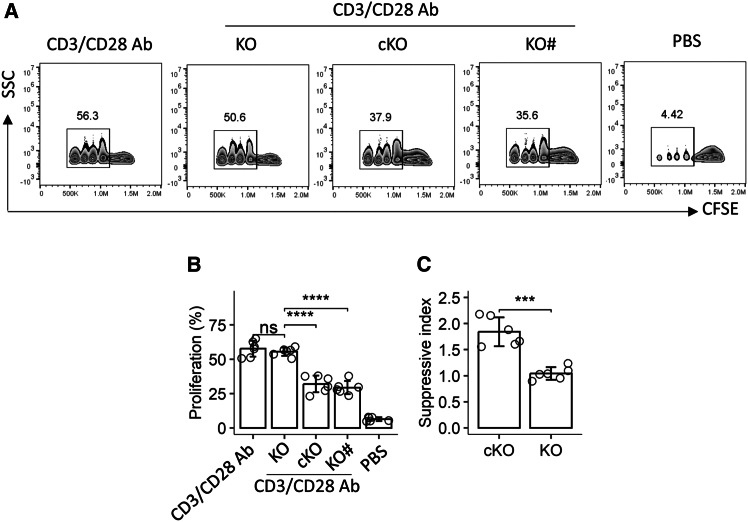
Tr1 cell-mediated suppression of effector T cell (Teff) proliferation requires KDM4A (A) Flow cytometry analysis of Teff proliferation (CFSE dilution) under different experimental conditions (each panel is denoted). (B) Quantification of proliferated Teffs (*n* = 6 mice/group). (C) Suppressive index of Tr1 cells calculated from proliferation data. Experimental groups: KO: knockout (Tr1 cells from *Kdm4a*^f/f^*Cd4Cre* mice). cKO: Tr1 cells from *Kdm4a*^f/f^ littermate controls. Teff: CD4^+^CD62L^+^ naive T cells activated with CD3/CD28 antibodies. KO#: CD4^+^CD25^+^CD127¯ Tregs isolated from KO mice. Data presentation: Bars represent mean ± SD. Individual dots indicate biological replicates. Statistical analysis: one-way ANOVA with Bonferroni post-hoc test. Significance: ∗∗∗∗*p* < 0.0001; ns = not significant.

Tr1 cells play a crucial role in maintaining immune homeostasis throughout the body. To investigate the impact of *Kdm4a* gene ablation on Tr1 cell function, we isolated Tr1 cells from the lungs of control *Kdm4a*^f/f^ (cKO) mice and *Kdm4a*^f/f^ Cd4Cre (KO) mice. These isolated Tr1 cells were then co-cultured with target effector CD4^+^CD62L^+^ T (Teff) cells in the presence of anti-CD3/CD28 antibodies for three days. Flow cytometric analysis revealed that while anti-CD3/CD28 antibody stimulation robustly induced Teff proliferation, this proliferation was significantly suppressed by the presence of Tr1 cells from cKO mice. In contrast, Tr1 cells isolated from *Kdm4a*-deficient KO mice exhibited only a marginal reduction in Teff proliferation (Figures 1A–1C). Notably, CD4^+^CD25^+^CD127^-^ Tregs from KO mice maintained their immunosuppressive effects on Teff proliferation (Figures 1A–1C). These findings indicate a specific impairment in the immunosuppressive function of Tr1 cells, but not Tregs, from *Kdm4a*-deficient mice.

### Spontaneous Th2 polarization in the airways of *Kdm4a*-deficient mice

**Figure 2:**
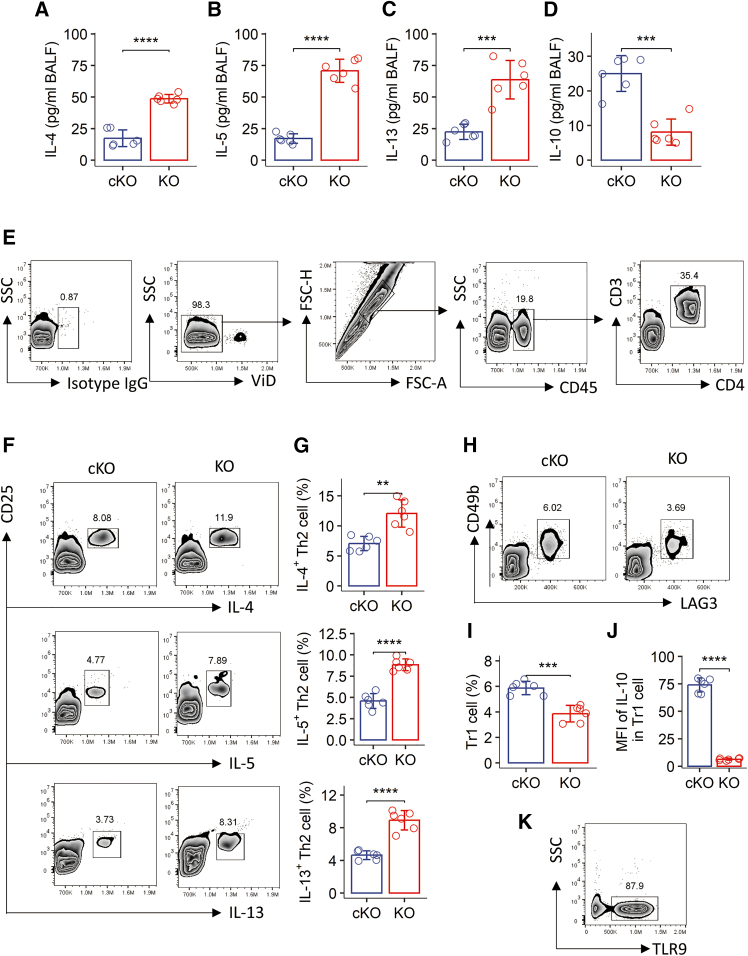
KDM4D deficiency in CD4^+^ T cells influences Th2 and regulatory T cell responses in the airways (A–D) Levels of indicated cytokines in bronchoalveolar lavage fluid (BALF) collected from *Kdm4d*^f/f^*Cd4*-Cre (KO) mice and *Kdm4d*^f/f^ (cKO) control mice. (E) Representative flow cytometry plots illustrating the gating strategy for the identification of CD3^+^CD4^+^ T cells from single-cell suspensions prepared from lungs. Dead cells and debris were excluded using viability dye (ViD), followed by sequential gating on CD45^+^, then CD3^+^, and finally CD4^+^ T cell populations. Isotype IgG staining results are also shown. (F) Representative flow cytometry plots illustrating the gating strategy to identify Th2 cells from the CD3^+^CD4^+^ T cell population. (G) Quantification of Th2 cell numbers in the lungs of KO and cKO mice. (H) Representative flow cytometry plots illustrating the gating strategy to identify Tr1 cells from the CD3^+^CD4^+^ T cell population. (I) Quantification of Tr1 cell numbers in the lungs of KO and cKO mice. (J) Mean fluorescence intensity (MFI) of IL-10 within the Tr1 cell population. (K) Representative flow cytometry plots illustrating the gating strategy to identify TLR9^+^ cells within the CD3^+^CD4^+^ T cell population. Data in bar graphs are presented as mean ± standard deviation (SD), with each individual dot representing one biological replicate. Statistical significance was determined by Student’s *t* test. ∗∗*p* < 0.01, ∗∗∗*p* < 0.001, ∗∗∗∗*p* < 0.0001. BALF, bronchoalveolar lavage fluid; KO, *Kdm4d*^f/f^*Cd4*-Cre mice (mice with Kdm4d-deficient CD4^+^ T cells); cKO, *Kdm4d*^f/f^ mice (control littermates); MFI, mean fluorescence intensity; ViD, viability Dye.

Given that Th2 polarization is a hallmark pathological feature of allergic diseases, we investigated whether *Kdm4a* gene ablation in CD4^+^ T cells affected allergic responses in the airways. Bronchoalveolar lavage fluid (BALF) was collected from cKO and KO mice and analyzed by ELISA. We observed a significant increase in Th2 cytokines (IL-4, IL-5, and IL-13) and a decrease in IL-10 levels in the BALF collected from KO mice compared to cKO mice (Figures 2A–2D). Furthermore, flow cytometric analysis of lung single-cell suspensions revealed a significantly higher frequency of Th2 cells in the airways of KO mice compared to cKO mice (Figures 2E–2G). While CD4^+^LAG3^+^CD49b^+^ Tr1 cells were detectable in both groups, their absolute numbers were significantly lower in KO mice (Figures 2H and 2I). Moreover, Tr1 cells from cKO mice exhibited detectable IL-10 expression, whereas Tr1 cells from KO mice showed only minimal IL-10 staining (Figure 2J). TLR9 was also detected in these Tr1 cell populations (Figure 2K). Together, these results demonstrate that KDM4A deficiency leads to a spontaneous Th2 polarization in the airways, characterized by altered cytokine profiles and reduced Tr1 cell numbers and IL-10 expression.

### Decreased *Il-10* expression in Tr1 cells correlates with airway Th2 polarization and impaired Tr1 immunosuppressive function

**Figure 3:**
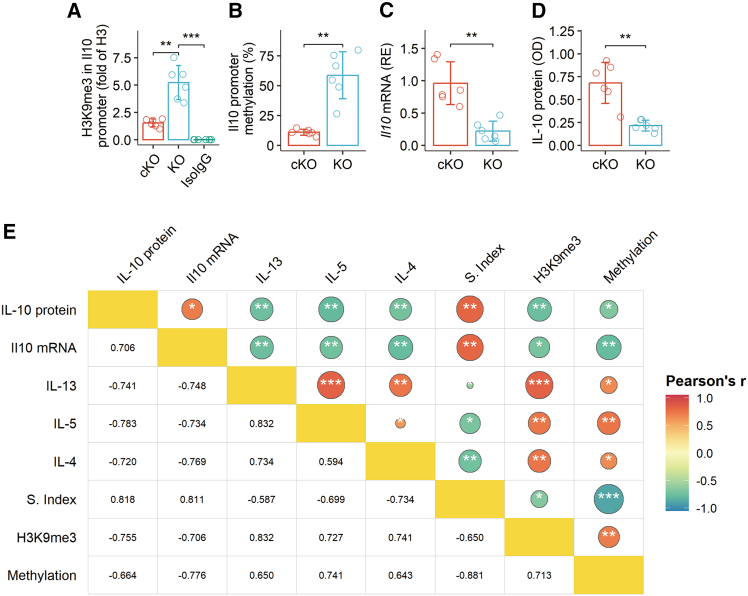
Correlation of IL-10 expression in airway Tr1 cells with airway Th2 polarization and their immunosuppressive function Airway Tr1 cells were isolated from mice, and their characteristics were analyzed by chromatin immunoprecipitation (ChIP), reverse transcription quantitative polymerase chain reaction (RT-qPCR), and enzyme-linked immunosorbent assay (ELISA). (A) Levels of H3K9me3 histone modification at the *Il-10* promoter locus. (B) Percentage methylation of the *Il-10* promoter. (C) Expression levels of *Il-10* mRNA. (D) Protein levels of IL-10. (E) A heatmap displaying correlation coefficients between indicated parameters. Data in bar graphs are presented as mean ± standard deviation (SD), with each individual dot representing one biological replicate. Statistical significance was determined by Student’s *t* test. ∗*p* < 0.05, ∗∗*p* < 0.01, ∗∗∗*p* < 0.001. IL.10.pr, IL-10 protein (as labeled in the figure); S.Index, suppressive index, representing the suppressive function of Tr1 cells (data presented in Figure 1C); KO, *Kdm4d*^f/f^*Cd4*-Cre mice; cKO, *Kdm4d*^f/f^ mice; IsoIgG, isotype IgG (control antibody used in ChIP).

IL-10 is widely recognized as the primary mediator by which Tr1 cells exert their immunoregulatory functions. Therefore, we proceeded to examine the status of *Il-10* expression in Tr1 cells from *Kdm4a*-deficient mice. Tr1 cells were isolated from the lungs of KO and cKO mice using flow cytometry cell sorting. Cellular extracts were prepared and analyzed by chromatin immunoprecipitation (ChIP), reverse transcription quantitative polymerase chain reaction (RT-qPCR), and ELISA. Our analysis revealed increased quantities of the repressive histone modification H3K9me3 at the *Il-10* promoter locus in Tr1 cells from KO mice (Figure 3A). Correspondingly, the *Il-10* promoter in Tr1 cells from KO mice exhibited a hypermethylated status (Figure 3B). This was accompanied by detectable decreases in both *Il-10* mRNA and IL-10 protein levels in Tr1 cells isolated from KO mice (Figures 3C and 3D). Correlation analysis demonstrated a positive correlation between KDM4A levels, *Il-10* expression in Tr1 cells, and their immunosuppressive functions. Conversely, a negative correlation was observed between the quantities of Th2 cytokines in BALF and *Il-10* expression in Tr1 cells (Figure 3E). These results suggest a strong association between the impaired *Il-10* expression in Tr1 cells and KDM4A deficiency. Given that Th2 polarization is a primary pathological feature of AA, these findings implicate that a reduction in KDM4A levels in Tr1 cells may contribute to the pathogenesis of AA.

### Decreased KDM4A at the *Il-10* promoter locus in Tr1 cells is associated with the airway allergic response

**Figure 4:**
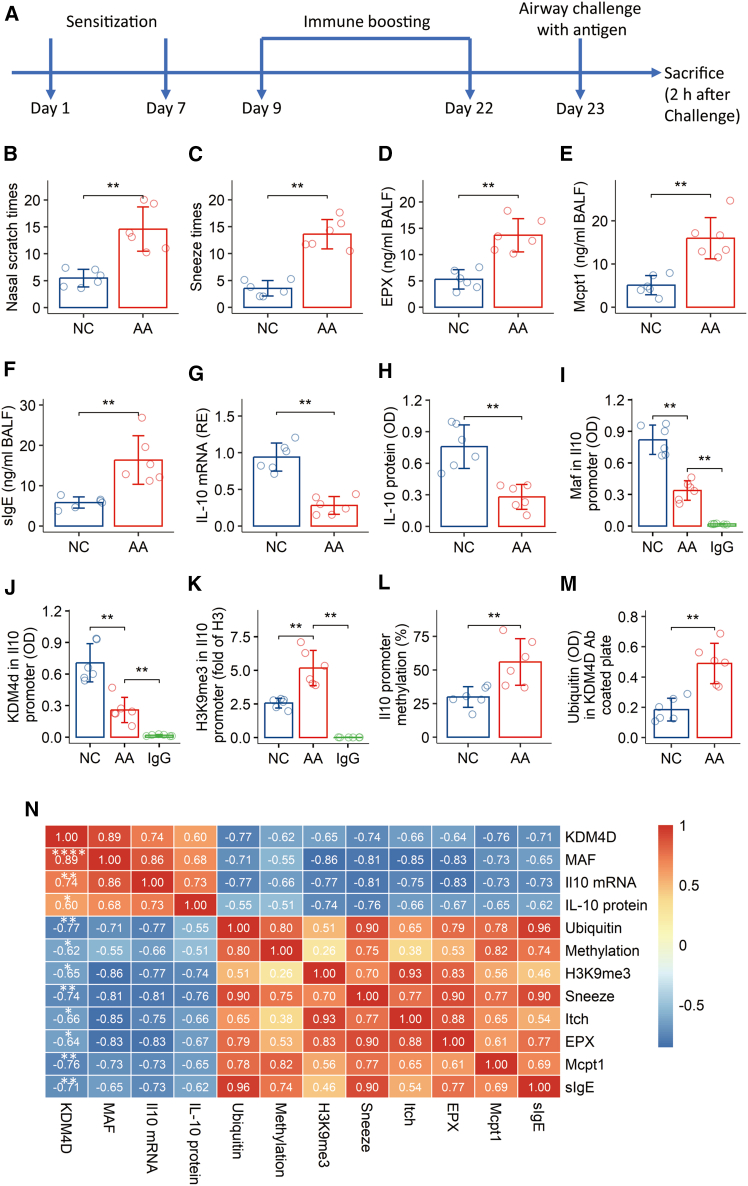
Airway Tr1 cell *Il-10* promoter methylation status correlates with airway allergic response (A) Schematic diagram illustrating the murine model of airway allergy (AA). (B–F) Assessment of various parameters of the airway allergic response. (G–H) Expression levels of IL-10 in airway Tr1 cells, including *Il-10* mRNA (G) and IL-10 protein (H). (I–K) Chromatin immunoprecipitation (ChIP) analysis of airway Tr1 cells showing the quantities of indicated factors at the *Il-10* promoter. (L) Methylation status of the *Il-10* promoter in airway Tr1 cells. (M) Quantification of ubiquitinated KDM4D protein. (N) Heatmap displaying correlation coefficients between the indicated items. Data in bar graphs are presented as mean ± standard deviation (SD), with each individual dot representing one biological replicate. Statistical significance for bar graph data (B–M) was determined by Student’s *t* test, and Pearson correlation coefficient test was used for the heatmap (N). ∗*p* < 0.05, ∗∗*p* < 0.01, ∗∗∗∗*p* < 0.0001. AA, airway allergy; NC, naive control; IgG, isotype IgG (control antibody used in ChIP).

As a demethylase, KDM4A is known to influence the expression of many genes. To investigate the status of KDM4A at the *Il-10* gene in Tr1 cells during AA, we established a murine model of AA with naive mice as controls (Figure 4A). As expected, these mice exhibited a robust AA response, characterized by symptoms like sneezing and increased quantities of allergic mediators and specific IgE in BALF (Figures 4B–4F). Tr1 cells isolated from the lungs of these AA mice displayed a significant decrease in IL-10 expression (Figures 4G and 4H). Furthermore, ChIP analysis of the *Il-10* promoter locus in airway Tr1 cells from AA mice showed reduced quantities of both Maf and KDM4A, alongside elevated quantities of H3K9me3 (Figures 4I–4K). Consistent with these epigenetic changes, the *Il-10* promoter was found to have a high level of hypermethylation (Figure 4L). Immunoblotting revealed that the KDM4A protein was in a hyper-ubiquitinated state in AA mice (Figure 4M). Correlation analysis demonstrated that the quantities of KDM4A at the *Il-10* promoter locus were positively correlated with IL-10 expression, but negatively correlated with the overall AA response and the methylation status of the *Il-10* promoter (Figure 4N). These results collectively indicate that sensitization during AA induces KDM4A ubiquitination and subsequent degradation. This, in turn, leads to hypermethylation of the *Il-10* promoter, ultimately compromising *Il-10* expression in Tr1 cells.

### Exogenous USP14 restores Tr1 cell immune regulatory functions by inducing KDM4A de-ubiquitination

**Figure 5:**
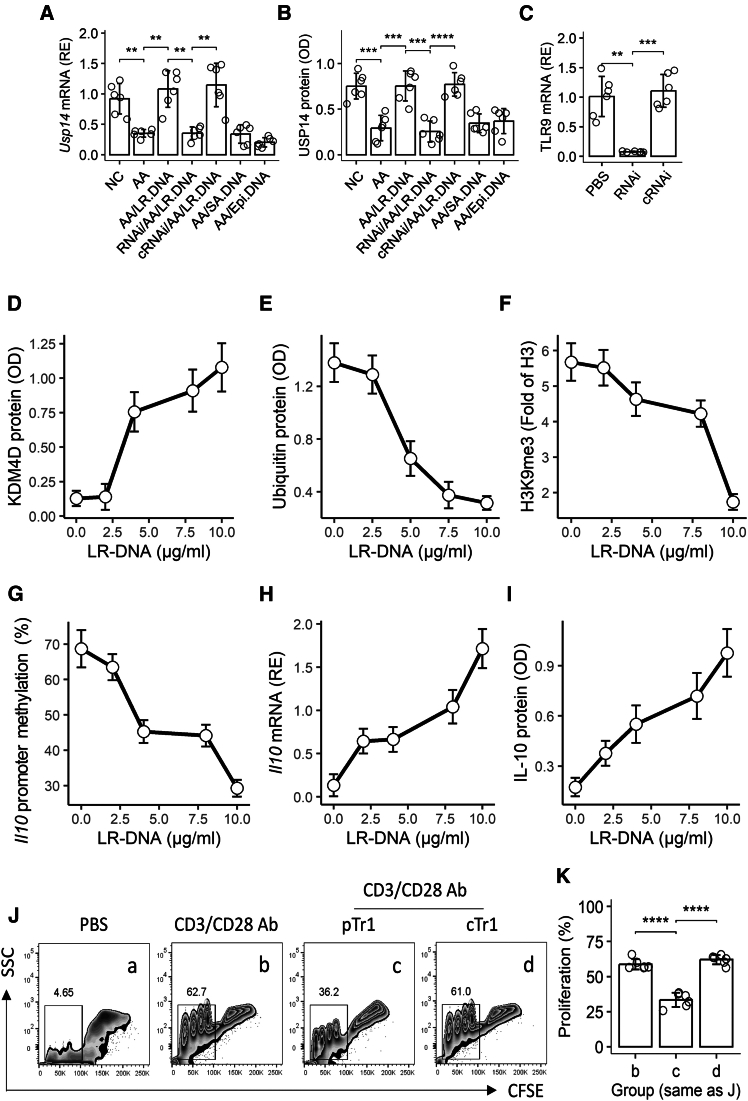
LR-DNA exposure restores immune suppressive function in airway Tr1 cells through epigenetic regulation of the Il-10 promoter (A and B) Usp14 expression in airway Tr1 cells following 24-h LR-DNA exposure (vs. control DNA). (A) *Usp14* mRNA levels (qRT-PCR). (B) USP14 protein quantification (ELISA; 10^6^ cells/sample). (C) Efficiency of *Tlr9* knockdown (*Tlr9* RNAi) in Tr1 cells (vs. control RNAi [cRNAi]). (D–F) Epigenetic modifications at the *Il-10* promoter. (D) KDM4D enrichment (ChIP-ELISA). (E) H3K9me3 levels (ChIP-ELISA). (F) KDM4D ubiquitination status. (G) Methylation frequency of the *Il-10* promoter (bisulfite sequencing). (H–I) *Il-10* expression in Tr1 cells. (H) *Il-10* mRNA (qRT-PCR). (I) IL-10 secretion (ELISA). (J–K) Functional suppression of effector T cell (Teff) proliferation by Tr1 cells. (J) Representative flow cytometry plots. *Ja*: Teffs + PBS. *Jb*: Teffs + CD3/CD28 Abs (positive control). *Jc*: Teffs + LR-DNA-treated AA Tr1 cells (pTr1). *Jd*: Teffs + untreated AA Tr1 cells (cTr1). (K) Quantification of proliferated Teffs (CFSE dilution). Experimental groups: NC: naive control mice. AA: airway allergy model. AA.DNA: AA Tr1 cells + LR-DNA. SA.DNA/Epi.DNA: control DNA from *Staphylococcus aureus* or mouse airway epithelial cells. Data presentation: Bars represent mean ± SD; individual dots = biological replicates (*n* ≥ 3/group). Statistics: One-way ANOVA with Bonferroni post-test. *∗∗p < 0.01, ∗∗∗p < 0.001, ∗∗∗∗p < 0.0001.* RNAi: TLR9 RNA interference. cRNAi: Control RNAi (cells were treated with negative control RNAi reagents, which contain scramble RNA sequence).

In the airway Tr1 cells of AA mice, we observed significantly reduced USP14 expression (Figures 5A and 5B). As USP14 is a known deubiquitinating enzyme,^20^ we hypothesized that USP14 supplementation might reverse the abnormal epigenetic state of KDM4A at the *Il-10* promoter in these cells.

To test this, we isolated Tr1 cells from AA mouse lungs and treated them with DNA from *Lactobacillus rhamnosus* (LR-DNA), based on published evidence of probiotic DNA’s immunomodulatory effects.^17^^,^^18^^,^^19^ After 24-h culture, LR-DNA (but not control DNA from *S. aureus* or airway epithelium) induced USP14 expression (Figures 5A and 5B). This effect was abolished by *Tlr9* knockdown (Figure 5C). Dose-response experiments revealed that LR-DNA treatment increased KDM4A enrichment at the *Il-10* promoter (ChIP-ELISA; Figure 5D); reduced KDM4A ubiquitination (Figure 5E); decreased H3K9me3 levels at *Il-10* (ChIP-ELISA; Figure 5F); and lowered *Il-10* promoter methylation (Figure 5G). Consequently, treated Tr1 cells showed elevated *Il-10* mRNA (Figure 5H) and IL-10 protein (Figure 5I), and enhanced suppression of Teff proliferation (Figures 5J and 5K). This demonstrates LR-DNA’s capacity to restore AA Tr1 cell function via the USP14-KDM4A-*Il-10* axis.

### Administration of LR-DNA attenuates experimental airway allergy

**Figure 6:**
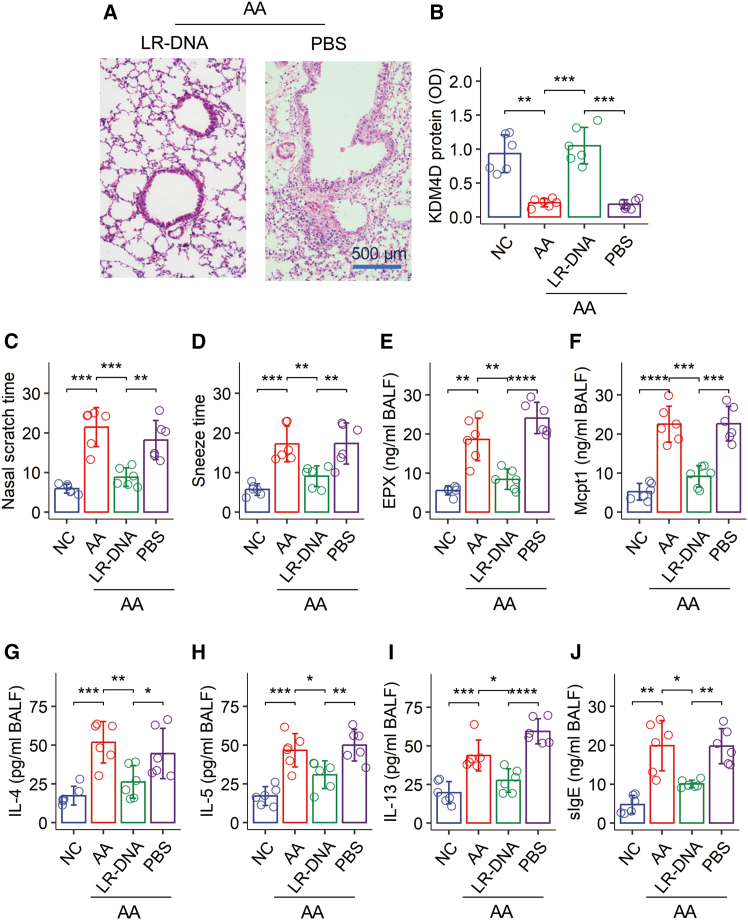
LR-DNA administration normalizes KDM4D status and mitigates experimental airway allergy In an established murine model of airway allergy (AA), a subset of AA mice was treated daily with LR-DNA for five days. (A) Representative histological images of lung tissue (original magnification: ×200; the bar is 500 μm). (B) Quantification of KDM4D at the *Il-10* promoter locus in isolated airway Tr1 cells. (C–J) Quantification of various parameters related to the airway allergic response. Data in bar graphs are presented as mean ± standard deviation (SD), with each individual dot representing one biological replicate. Statistical significance was determined by one-way ANOVA followed by Bonferroni’s (or post-hoc) test. ∗*p* < 0.05, ∗∗*p* < 0.01, ∗∗∗*p* < 0.001, ∗∗∗∗*p* < 0.0001. AA, airway allergy; NC, naive control.

Given the established immune regulatory functions of probiotic DNA, we prepared probiotic DNA from *Lactobacillus rhamnosus*, designated as LR-DNA, to investigate its potential therapeutic effects on AA. A murine model of AA was established, and the characteristic allergic responses were confirmed by parameters such as significant lung inflammation (Figure 6A), observable clinical symptoms (e.g., sneezing), increased levels of allergic mediators (EPX and Mcpt1), elevated Th2 cytokines (IL-4, IL-5, and IL-13), and increased specific IgE (sIgE) in BALF (Figures 6C–6J).

Daily nasal instillation of LR-DNA for five days significantly ameliorated these AA responses. Specifically, LR-DNA treatment reduced lung inflammation (Figure 6A) and notably increased the quantity of KDM4D at the *Il-10* promoter locus in airway Tr1 cells (Figure 6B). Overall, the exacerbated AA symptoms and inflammatory markers (Figures 6C–6J) were significantly attenuated.

**Figure 7:**
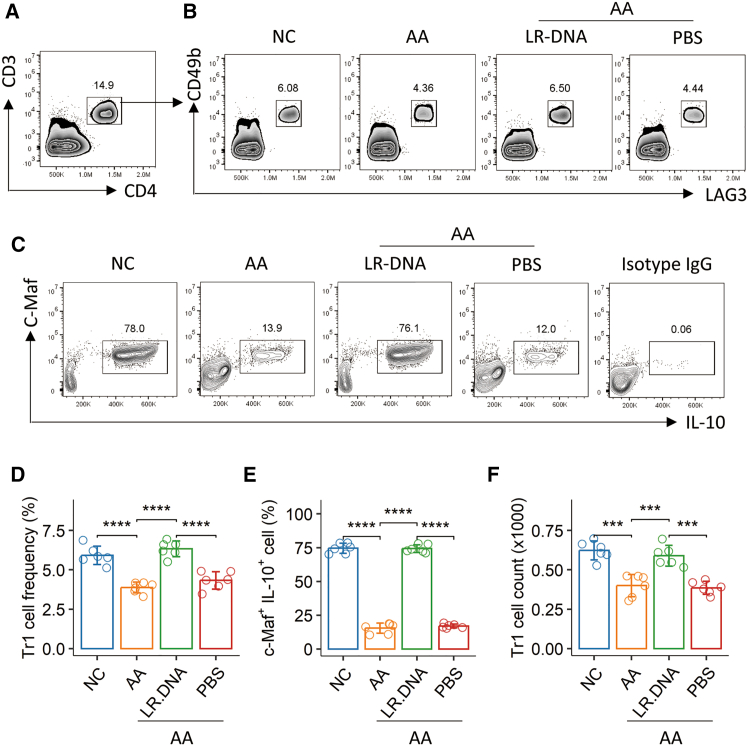
LR-DNA modulates regulatory T cell (Tr1) populations in the airways of airway allergy mice Single-cell suspensions were prepared from lung tissues and analyzed by flow cytometry (10^5^ cells per sample). (A) Representative flow cytometry plots illustrating the gating strategy for CD3^+^CD4^+^ T cells. (B) Representative flow cytometry plots illustrating the gating strategy for LAG3^+^CD49b^+^ Tr1 cells, identified from the CD3^+^CD4^+^ T cell population. (C) Representative flow cytometry plots illustrating the gating strategy for c-Maf^+^IL-10^+^ cells, identified within the Tr1 (LAG3^+^CD49b^+^) population. (D–F) Bar graphs showing the frequency of total Tr1 cells (LAG3^+^CD49b^+^) (D), the frequency of CD3^+^CD4^+^c-Maf^+^IL-10^+^ Tr1 cells (E), and the absolute number of Tr1 cells (F) in the lungs. Data in bar graphs are presented as mean ± standard deviation (SD), with each individual dot representing one biological replicate. Statistical significance was determined by one-way ANOVA followed by Bonferroni’s post-hoc test. ∗∗*p* < 0.01, ∗∗∗*p* < 0.001, ∗∗∗∗*p* < 0.0001. AA, airway allergy; NC, naive control.

Furthermore, LR-DNA administration significantly increased both the frequency and absolute numbers of Tr1 cells in the BALF samples of AA mice (Figures 7A, 7B, 7D, 7F). In AA mice, the frequency of *c*-Maf (the *Il-10* transcription factor)^+^IL-10^+^ cells within the Tr1 population was significantly reduced compared to naive control mice. This reduction was reversed by LR-DNA administration, which increased the frequency of these *c*-Maf^+^IL-10^+^ Tr1 cells (Figures 7C and 7E). Collectively, these results demonstrate that LR-DNA administration effectively mitigates experimental AA and restores key aspects of Tr1 cell function and numbers in the airways.

## Discussion

Current data show a decrease in KDM4A in the *Il-10* promoter of airway Tr1 cells. The reduction of KDM4A results in increased H3K9me3 (a repressive histone modification) in the *Il-10* promoter and hypermethylation of the *Il-10* promoter. As a consequence, the expression of *Il-10* in Tr1 cells is hindered, which compromises their immune suppressive function. We demonstrate that upregulation of USP14 by LR-DNA restores the immune regulatory function of airway Tr1 cells. Ultimately, administration of LR-DNA mitigates experimental AA.

Our data show that ablation of the *Kdm4a* gene in Tr1 cells resulted in spontaneous Th2 polarization in the airways. Th2 polarization is the primary pathological mechanism for AA. The AA response is one of the main parameters used to judge the therapeutic effects of immunotherapy in AA subjects,^21^^,^^22^ and it is also used to verify the successful establishment of AA animal models.^23^^,^^24^ While Th2 polarization has been extensively studied, its causal mechanisms have not been fully understood. Previous studies also found that abnormal states of Tr1 cells were associated with allergic diseases.^25^ Our current data reveal that KDM4A deficiency in Tr1 cells is an important factor associated with Th2 polarization.

Tr1 cells are a crucial fraction of the major immune regulatory cells. By producing immune regulatory cytokines, such as IL-10, Tr1 cells suppress the activities of other immune cells.^26^ Our current data show a decrease in the expression of *Il-10* in airway Tr1 cells from AA mice. Through statistical analysis, we detected a correlation between the *Il-10* expression in airway Tr1 cells and the AA response. These data indicate the importance of *Il-10* expression in airway Tr1 cells in the context of the AA response. Thus, identifying the causal factors that compromise *Il-10* expression in Tr1 cells is imperative.

It is known that the methylation status is an indicator of gene transcription.^5^ Our data indicate a hypermethylation status in the *Il-10* promoter locus and elevated repressive histone H3K9me3 marks in the surrounding region of airway Tr1 cells from AA mice. This demonstrates that the transcription of the *Il-10* gene is inactive. The occurrence of this phenomenon, involving progressive DNA methylation at relevant CpG regions, leads to a repressive *Il-10* gene expression program in Tr1 cells, as previously reported.^27^ Prompted by previous studies,^27^ which show that KDM4A is a crucial factor in maintaining active gene transcription, we checked the quantities of KDM4A at the *Il-10* promoter. Our findings indicate a decrease in the quantity of KDM4A in the *Il-10* promoter of airway Tr1 cells. This could explain the abnormal epigenetic profile in the *Il-10* promoter of Tr1 cells in AA mice. Increasing the quantity of KDM4A is therefore expected to upregulate the transcription of its targeted genes.

Our data show hyper-ubiquitination of the KDM4A protein in the *Il-10* promoter locus of airway Tr1 cells from AA mice. Ubiquitination is a major pathway for protein degradation. Many studies show that hyper-ubiquitination hinders gene transcription by inducing transcription factor dysfunction.^28^^,^^29^^,^^30^ Current data show an inverse relationship between the quantity of KDM4A and the amount of ubiquitin at the *Il-10* promoter locus. Such a phenomenon was also reported by previous studies. Liu et al. indicate that a decrease in KDM4A can be found at the *IL-12* and *IL-23* gene loci, which reduces the expression of IL-12 and IL-23.^31^ Our data demonstrate that KDM4A is required for maintaining the methylation status of the *Il-10* gene in Tr1 cells at proper levels.

The *Il-10* promoter in airway Tr1 cells of AA mice showed a reduction in USP14 quantity, as indicated by our data. USP14 is an enzyme responsible for de-ubiquitination.^20^ Its reduction can result in inadequate de-ubiquitination for KDM4A.^31^ Consequently, the amount of KDM4A can be decreased. This implies that increasing the amount of USP14 may increase the quantity of KDM4A through a de-ubiquitination mechanism. Previous studies also showed that KDM4A plays a role in stabilizing the expression of *IL-12* and *IL-23* genes in dendritic cells. When TLR4 is activated, Trabid, a de-ubiquitinase, is recruited to decrease the ubiquitination status of KDM4A, thereby maintaining the expression of IL-12 and IL-23.^32^ Our data show a similar phenomenon. Exposure to LR-DNA, a probiotic DNA, activated TLR9 to recruit USP14 in Tr1 cells. This resulted in an increase in the amount of KDM4A at the *Il-10* promoter locus of airway Tr1 cells isolated from AA mice. Importantly, this restored the immune suppressive function of the Tr1 cells.

We tested the hypothesis that administration of LR-DNA can recruit USP14 to suppress experimental AA by modulating the epigenetic status of Tr1 cells. The AA response, encompassing clinical symptoms, allergic mediators, Th2 cytokines, and sIgE in BALF, was effectively reduced by daily nasal instillations of LR-DNA for five days. Tr1 cell dysfunction has also been found in other immune disorders, such as food allergy,^33^^,^^34^ atopic dermatitis,^35^ allergic rhinitis,^36^ arthritis,^37^ and systemic lupus erythematosus.^38^ Therefore, investigating whether LR-DNA also improves the immune regulatory functions of Tr1 cells in these immune disorders is a worthwhile future direction. The data suggest that the administration of LR-DNA has translational potential for the treatment of AA and other immune inflammatory disorders.

### Limitation of this study

All the data in this study were generated from animal samples. Since data from animal models may not be perfectly comparable with human beings, further studies using human samples for similar investigation may be necessary.

## Resource availability

### Lead contact

Further information and requests for resources and reagents should be directed to and will be fulfilled by the lead contact: Pingchang Yang (pcy2356@163.com).

### Materials availability

All new reagents are available in our lab for non-commercial research.

### Data and code availability

• All the data are included in this paper.
• This paper does not have specific code.
• Any additional information required to reanalyze the data reported in this paper is available from the lead contact upon request.

## Acknowledgments

This study was supported by research grants of the 10.13039/501100001809National Natural Science Foundation of China (32090052, 82371122), Shenzhen Key Medical Discipline Construction fund (SZXK062), the 10.13039/501100003453Natural Science Foundation of Guangdong Province (2022A1515012617), Shenzhen Science and Technology Innovation Bureau (JCYJ20240813143215019), and 10.13039/501100002858China Postdoctoral Science Foundation (2023M740837).

## Author contributions

Huanping Zhang, X.L., M.Z., X.C., L.M., L.L., Hanqing Zhang, G.W., and Q.H. performed experiments, analyzed data, and reviewed manuscript. Y.L. and P.Y. supervised experiments. P.Y. and H Zhang designed the project. P.Y. organized the study and prepared the manuscript.

## Declaration of interests

The authors declare no competing interests.

## STAR★Methods

### Key resources table

**Table:** 

REAGENT or RESOURCE	SOURCES	IDENTIFIER
**Antibodies**		

CD28	Santa Cruz Biotech	sc-70612
CD3	Santa Cruz Biotech	sc-20047, AF488
CD4	Santa Cruz Biotech	sc-19641, AF546
CD25	Santa Cruz Biotech	sc-393326, AF648
CD62L	Santa Cruz Biotech	sc-390756, AF700
CD127	Santa Cruz Biotech	sc-514445, AF488
IL-4	Santa Cruz Biotech	sc-53084, AF-594
IL-5	Santa Cruz Biotech	sc-398334, AF648
IL-13	Santa Cruz Biotech	sc-393365, AF680
LAG3	Santa Cruz Biotech	sc-514993, AF680
CD49b	Santa Cruz Biotech	sc-74466, AF594
KDM4A	Santa Cruz Biotech	sc-373850
IL-10	Santa Cruz Biotech	sc-32815
c-Maf	Santa Cruz Biotech	sc-293420

**Critical commercial assays**		

EPX	R&D System	MEP00B
Mcpt1	R&D System	MJE00B
IL-4	R&D System	M4000B
IL-5	R&D System	M5000
IL-10	R&D System	M1000B
IL-13	R&D System	M1300CB
Ubiquitin	AmyJet	GBS-IT13866
H3	AmyJet	A-QEK07225
KDM4A	HSA Biotech	HAS-47761
H3K9me3	EpigenTek	P-3118-48
DME-sIgE	Chemical Book	CB85808579
USP14	Weikscience	N/A
Amine reactive dyes (ViD)	Miltenyi Biotech	130-110-207

**Software and algorithms**		

R v4.3.1	R Development Core Team, 2022	https://mirrors.bfsu.edu.cn/CRAN/
FlowJo v10	BD	https://www.flowjo.com/
BD FACSCanto™ Clinical Software	BD	https://www.bdbiosciences.com/en-eu/products/software/instrument-software/bd-facscanto-clinical-software

### Experimental model and subject details

#### Ethical statement

The animal ethics committee at our university gave their approval to the animal experiments with the approval number of A202300080. ARRIVAL’s guidelines were followed in conducting all animal experiments.

#### Mice

Six to eight-week-old male BALB/c mice were acquired from the Guangdong Experimental Animal Center (Fushan, China). *Kdm4a* floxed (*Kdm4a*^f/f^) mice and CD4-Cre (*Cd4Cre*) mice were originally purchased from Jackson Laboratory (Bar Harbor, ME). Conditional *Kdm4a* knockout in CD4^+^ T cells (*Kdm4a*ΔCD4) was achieved by breeding *Kdm4a*^f/f^ mice with *Cd4Cre* mice. Mice used in experiments were from at least the fifth generation, and the successful conditional gene knockout was systematically confirmed prior to experimentation. All mice were housed under specific pathogen-free (SPF) conditions with *ad libitum* access to food and water. All animal experiments were approved by the institutional Animal Ethics Committee (Approval No. A202300080) and strictly adhered to the ARRIVE guidelines.

### Method details

#### Establishment of an airway allergy (AA) mouse model

The allergic airway inflammation (AA) mouse model was established in 6–8-week-old male mice following previously published protocols.^3^^,^^39^ Mice were sensitized via subcutaneous injection of dust mite extract (DME, 0.1 mg/mouse in 0.1 mL alum) on the back on days 1 and 7. Following sensitization, mice received daily intranasal instillations of DME (5 mg/mL, 20 μL/nostril) from day 9 to day 22. On day 23, a final large-dose challenge was administered via intranasal instillation (50 mg/mL, 20 μL/nostril). Mice were sacrificed by cervical dislocation 3 h post-challenge for subsequent analysis.

#### Evaluation of AA response

The severity of the AA response was assessed based on eight key parameters: clinical symptoms (nasal itch and sneezing were counted during 30 min after challenge), levels of allergic mediators (eosinophil peroxidase [EPX] and mouse mast cell protease-1 [Mcpt1]), Th2 cytokines (IL-4, IL-5, and IL-13), and specific IgE (sIgE) in bronchoalveolar lavage fluid (BALF).

#### Enzyme-linked immunosorbent assay (ELISA)

Protein concentrations in BALF, cell culture supernatants, and cellular protein extracts were quantified using commercial ELISA kits according to the manufacturers’ instructions.

#### Preparation of single cells from the lungs

Following sacrifice, lungs were carefully excised, minced into small pieces, and enzymatically digested in a solution containing collagenase IV (0.5 mg/mL) and DNase I (0.2 mg/mL) at 37°C for 30 min with gentle agitation. The resulting cell suspension was then filtered through a cell strainer to obtain single-cell suspensions for downstream experiments.

#### Cell culture

Cells were cultured in RPMI-1640 medium supplemented with 10% fetal calf serum (FCS), 100 U/mL penicillin, 0.1 mg/mL streptomycin, and 2 mM L-glutamine. Cell viability, assessed by Trypan Blue exclusion assay, consistently ranged between 97% and 99%.

#### Flow cytometry

Live cells were stained for surface and/or intracellular markers with fluorochrome-conjugated antibodies, or corresponding isotype controls, as described previously.^39^ Data acquisition was performed using a BD FACSCanto II flow cytometer. Analysis was conducted using FlowJo software, with isotype IgG staining serving as the gating reference.

#### Isolation of Tr1 cells

Lung single-cell suspensions were stained with fluorochrome-conjugated antibodies against CD3, CD4, LAG3, and CD49b. Tr1 cells (CD3^+^CD4^+^LAG3^+^CD49b^+^) were then sorted using a BD FACSAria flow cytometer. The purity of sorted populations was verified by post-sort flow cytometry, and sorting was repeated if purity did not exceed 90%.

#### Isolation of naive CD4^+^ T cells

Splenic single-cell suspensions were stained with fluorochrome-conjugated antibodies against CD3, CD4, and CD62L. Naive CD4^+^ T cells (CD3^+^CD4^+^CD62L^+^) were sorted using a BD FACSAria flow cytometer. The purity of sorted populations was verified by post-sort flow cytometry, and sorting was repeated if purity did not exceed 90%.

#### Assessment of the immune suppressive effects of Tr1 cells

The immunosuppressive capacity of sorted Tr1 cells was assessed in co-culture assays with CFSE-labeled naive CD4^+^ T cells (Teffs, identified as CD4^+^CD62L^+^ cells) at a ratio of 1:9 (Tr1:Teff) in the presence of anti-CD3/CD28 antibodies (5 μg/mL each). After 3 days of co-culture, Teff proliferation was quantified by CFSE dilution using flow cytometry, serving as a measure of Tr1 cell-mediated suppression.

#### Real-time quantitative RT-PCR (RT-qPCR)

Total RNA was extracted from harvested cells using a Qiagen RNA extraction kit and reverse-transcribed into cDNA using a Qiagen reverse transcription kit. Real-time quantitative PCR (RT-qPCR) was performed on a Bio-Rad CFX96 qPCR device using SYBR Green Master Mix. Primer sequences were as follows: *Kdm4a* (forward: tcaccgatttatggtgctga, reverse: ggtcttccacatgccaaagt), *Il10* (forward: ccaagccttatcggaaatga, reverse: ttttcacaggggagaaatcg), and *Usp14* (forward: cgttctgtgcctgaactcaa, reverse: aaaggccatgtgcagaaact). Relative gene expression was calculated using the 2-ΔΔCt method, normalized to the housekeeping gene *Actb* (forward: agccatgtacgtagccatcc, reverse: ctctcagctgtggtggtgaa).

#### Chromatin immunoprecipitation (ChIP)

Tr1 cells (1 x 10^4^ cells/sample) were isolated from lung single-cell suspensions as described previously. Cells were fixed with 1% formaldehyde for 15 min, then lysed in RIPA buffer. Chromatin was sonicated to yield DNA fragments of approximately 200–500 bp. Pre-clearing was performed by incubating chromatin with Protein G beads, which were subsequently discarded. Chromatin was then incubated overnight with 0.5 μg/mL of anti-*c*-Maf antibody. Immune complexes were captured by Protein G beads, washed extensively, and eluted. For DNA analysis, extracted DNA was quantified by qPCR using primers specific for the *Il10* promoter (forward: ccgggagtgtaccctctaca, reverse: tcagtttgggtgggaagaac). For protein analysis, the protein portion of the immune complexes was subjected to ELISA or 'crossing ELISA' (described below) to quantify levels of c-Maf, KDM4A, USP14, and ubiquitin, respectively.

#### Measurement of ChIP-derived proteins using crossing ELISA

A 'crossing ELISA' was performed as previously described^19^ to quantify specific proteins associated with immunoprecipitated complexes. Taking ubiquitin detection as an example: Microplates were coated overnight with 1 μg/mL anti-KDM4A antibody, then blocked with 1% bovine serum albumin (BSA) for 30 min. HRP-conjugated anti-ubiquitin antibody (200 pg/mL, BiossAntibiotics) was added and incubated for 2 h. Color development was achieved by adding OPD buffer (1 mg/mL containing 1% H_2_O_2_) for 20 min, followed by stopping the reaction with 1 M H_2_SO_4_. Absorbance was read at 496 nm using a microplate reader.

#### Methylation specific PCR (msPCR)

Methylation-specific PCR (msPCR) was performed on genomic DNA extracted from sorted Tr1 cells using a Qiagen DNA extraction kit. DNA (1 μg) underwent sodium bisulfite conversion using the EZ DNA Methylation kit (Zymo Research, Orange, CA) according to the manufacturer’s protocol, following established procedures.^40^ msPCR was then performed on a Bio-Rad CFX96 qPCR device with SYBR Green Master Mix to target the methylated and unmethylated regions of the *Il10* promoter. Primers used were: Unmethylated *Il10* (forward: gaggtttgaagaaaattagtttttt, reverse: aaaccctcatctataaattccattc) and Methylated *Il10* (forward: ggtttgaagaaaattagttttttcg, reverse: ctccactcaacctaaattaaacgtc). Results were processed using the 2^−ΔΔCt^ method and presented as the percentage of *Il10* promoter methylation.

#### Preparation of DNA

Genomic DNA was extracted from three sources: *Lactobacillus rhamnosus* GG (ATCC 53103), *Staphylococcus aureus* (ATCC 6538), and mouse airway epithelial cells. For bacterial strains, 1 mL overnight cultures were centrifuged at 8,000 × g for 5 min to pellet cells. Mouse airway epithelial cells were harvested by trypsinization and washed twice with PBS. DNA extraction was performed using the QIAamp Fast DNA Mini Kit (Qiagen, Hilden, Germany) following the manufacturer’s protocol with slight modifications for bacterial samples: bacterial pellets were resuspended in 180 μL of Buffer ATL supplemented with 20 μL proteinase K (20 mg/mL) and incubated at 56°C for 30 min to ensure complete lysis. DNA quantity and purity were determined spectrophotometrically using a Multiskan GO microplate spectrophotometer (Thermo Fisher Scientific, Waltham, MA, USA), measuring absorbance at 260 nm (for concentration) and the A260/A280 ratio (for purity). Only DNA samples with A260/A280 ratios ranging from 1.8 to 2.0 were used for subsequent experiments, ensuring high purity. The extracted DNA samples were designated as: LR-DNA (*L. rhamnosus* GG DNA), SA-DNA (*S. aureus* DNA), and Epi-DNA (mouse airway epithelial cell DNA).

#### Histology of the lung

Excised lung tissues were fixed in 4% formalin, embedded in paraffin, and sectioned. Tissue sections were then stained with hematoxylin and eosin (H&E) and observed under a light microscope for histological evaluation.

#### RNA interference (RNAi)

For RNA interference (RNAi), single cells were transfected with specific shRNA plasmids or control scramble shRNA plasmids using Lipofectamine 3000 according to the manufacturer’s protocol. RNAi knockdown efficiency was evaluated 2 days post-transfection by RT-qPCR.

### Quantification and statistical analysis

Statistical analyses were performed using R4.5.1 software. Differences between two groups were determined by Student’s *t* test. For multiple comparisons, one-way ANOVA followed by Bonferroni’s *post-hoc* test was employed. Correlations between variables were assessed using Pearson’s correlation coefficient. A *p*-value <0.05 was considered statistically significant.
